# Factors Associated with Mortality among Hospitalized Patients with COVID-19: A Retrospective Cohort Study

**DOI:** 10.4269/ajtmh.20-1170

**Published:** 2020-11-17

**Authors:** Paulo Ricardo Martins-Filho, Adriano Antunes de Souza Araújo, Luciana Xavier Pereira, Lucindo José Quintans-Júnior, Waneska de Souza Barboza, Taise Ferreira Cavalcante, Mércia Feitosa de Souza, Marco Aurélio de Oliveira Góes, Victor Santana Santos

**Affiliations:** 1 Federal University of Sergipe, Sao Cristovao, Brazil;; 2 Federal University of Alagoas, Arapiraca, Brazil;; 3 Aracaju City Hall, Municipal Health Secretariat, Aracaju, Brazil;; 4 Government of Sergipe State, State Health Secretariat, Aracaju, Brazil

## Abstract

Information on the risk factors for COVID-19 mortality in low- and middle-income countries is still scarce. In this retrospective cohort study, we analyzed the factors associated with COVID-19 mortality in hospitalized patients in a poor area of Brazil. Logistic regression was used to identify factors independently associated with mortality, including gender, age, and the presence of underlying medical conditions. A total of 1,207 patients were included in the analysis, and a 1.5-fold increase in COVID-19 mortality was found among patients aged > 65 years with hypertension and diabetes (odds ratio [OR]: 1.50, 95% CI: 1.02–2.19). Moreover, infectious disease (OR: 4.31, 95% CI: 1.39–13.39), kidney disease (OR: 2.59, 95% CI: 1.27–5.27), and heart disease (OR: 2.00, 95% CI: 1.31–3.04) were also predictive for COVID-19 in-hospital death. This large cohort provides important data on potential factors associated with COVID-19 mortality in Brazil.

## INTRODUCTION

SARS-CoV-2 is an emerging RNA virus associated with a severe acute respiratory disease known as COVID-19. Globally, SARS-CoV-2 infected more than 50 million people and caused more than 1.2 million deaths until November 9, 2020. In high-income countries, COVID-19 mortality has been higher in men, older people, and those with some comorbidity, including hypertension, diabetes, and cardiovascular disease.^1^ However, information on the risk factors in low- and middle-income countries is still scarce. Herein, we report the factors associated with COVID-19 mortality in hospitalized patients in a poor area of Brazil.

## METHOD

This retrospective cohort study enrolled all adults with PCR-confirmed COVID-19 who were consecutively admitted to public and private hospitals in Aracaju, Sergipe state, Northeast Brazil, from March 17 to July 14, 2020. Aracaju is a coastal city located in the poorest region in a country marked by large regional disparities. This city has an estimated population of 657,053 inhabitants and a high proportion of low-income households (42.9%). Recently, it was found that neighborhoods with poor living conditions in Aracaju are more likely to have higher COVID-19 fatality rates.^2^

Data on COVID-19 cases and deaths were extracted from the microdata catalog and official bulletin of the Municipal Secretariat of Health. We excluded patients with incomplete information, pregnant women, patients aged 18 years and younger, and those treated in hospital outside of Aracaju. Data collected included the outcome of interest (death) and potential factors associated with COVID-19 mortality: gender, age, and the presence of underlying medical conditions (gastrointestinal disease, chronic pulmonary disease, asthma, neurodegenerative disease, stroke, hypothyroidism, cancer, non-HIV immunosuppressive disease, obesity, infectious disease, kidney disease, heart disease, hypertension, and diabetes).

Data were presented as absolute and relative frequencies, and comparisons between survivors and non-survivors were expressed as odds ratio (OR) with 95% CIs. Logistic regression was used to identify factors independently associated with mortality. First, a univariate model was performed. Afterward, interaction was checked to control the relationship between covariates and their effect on the outcome. Finally, we included in multivariate analyses with backward selection all factors that have shown a relaxed *P*-value ≤ 0.30. Two multivariate models were developed. Model 1 included a standard logistic regression, and model 2 incorporated interaction for age, hypertension, and diabetes. *P*-values < 0.05 were considered statistically significant. All data were de-identified, and analyzes were performed using JASP software version 0.13 (JASP Team, Amsterdam, Netherlands).

## RESULTS

A total of 1,207 of 1,632 patients initially considered met the eligibility criteria and were included in the analysis. The median (IIQ) age was 60 (46–73) years. Seven hundred twenty-four (60.0%) were men, and 708 (58.7%) patients had at least one coexisting condition (Table 1).

**Table 1:** Results of univariate logistic regression analysis of association between clinical factors and death in patients with COVID-19

Covariates	Survivors (*n* = 854), *n* (%)	Non-survivors (*n* = 353), *n* (%)	Odds ratio	95% CI	*P*-value*
Gender (male)	513 (60.1)	211 (59.8)	0.99	0.77–1.27	0.924
Age > 65 years	283 (33.1)	203 (57.5)	2.73	2.12–3.52	< 0.001
Gastrointestinal disease	11 (1.3)	3 (0.8)	0.66	0.18–2.37	0.521
Chronic pulmonary disease	28 (3.3)	12 (3.4)	1.04	0.52–2.07	0.915
Asthma	11 (1.3)	3 (0.8)	0.66	0.18–2.37	0.521
Neurodegenerative disease	17 (2.0)	9 (2.5)	1.29	0.57–2.92	0.544
Stroke	28 (3.3)	14 (4.0)	1.22	0.63–2.34	0.554
Hypothyroidism	14 (1.6)	6 (1.7)	1.04	0.40–2.72	0.940
Cancer	28 (3.3)	14 (4.0)	1.22	0.63–2.34	0.554
Non-HIV immunosuppressive disease	10 (1.2)	7 (2.0)	1.71	0.65–4.52	0.282
Obesity	77 (9.0)	23 (6.5)	0.70	0.43–1.14	0.153
Infectious disease	5 (0.6)†	8 (2.3)‡	3.94	1.28–12.12	0.017
Kidney disease	15 (1.8)	18 (5.1)	3.00	1.50–6.03	0.002
Heart disease	56 (6.6)	46 (13.0)	2.14	1.42–3.22	< 0.001
Hypertension	308 (36.1)	143 (40.5)	1.21	0.94–1.56	0.137
Diabetes	209 (24.5)	102 (28.9)	1.25	0.95–1.66	0.110

* Variables with relaxed *P*-value ≤ 0.30 were included in the multivariate analysis with backward selection.

† Survivors with infectious disease (HIV: 1, visceral leishmaniasis: 1, tuberculosis: 1, bacterial infection: 2).

‡ Non-survivors with infectious disease (HIV: 3, lepromatous leprosy: 4, tuberculosis: 1).

Overall, 353 patients (29.2%) died. The median age of survivors was lower than that of non-survivors (median [IIQ]: 57 [43–70] versus 68 [55–79]; *P* < 0.001). The univariate analysis identified eight covariates as candidates for the multivariate model, including age > 65 years, non-HIV immunosuppressive disease, obesity, infectious disease, kidney disease, heart disease, hypertension, and diabetes (Table 1).

The standard multivariate regression analysis showed that COVID-19 mortality was significantly associated with age > 65 years (OR: 2.76, 95% CI: 2.12–3.60), following underlying medical conditions: infectious disease (OR: 5.26, 95% CI: 1.66–16.71), kidney disease (OR: 2.60, 95% CI: 1.24–5.42), and heart disease (OR: 1.67, 95% CI: 1.08–2.57). By adding interactions, we found that mortality was increased 1.5-fold among patients aged > 65 years with hypertension and diabetes (OR: 1.50, 95% CI: 1.02–2.19). In this model, infectious disease (OR: 4.31, 95% CI: 1.39–13.39), kidney disease (OR: 2.59, 95% CI: 1.27–5.27), and heart disease (OR: 2.00, 95% CI: 1.31–3.04) remained predictive for COVID-19 in-hospital death (Table 2).

**Table 2:** Results of the multivariate logistic regression analyses

Covariates	Standard analysis		Interaction analysis	
	OR (95% CI)	*P*-value	OR (95% CI)	*P*-value
Age > 65 years	2.76 (2.12–3.60)	< 0.001	–	*
Non-HIV immunosuppressive disease	–	NS	–	NS
Obesity	–	NS	–	NS
Infectious disease	5.26 (1.66–16.71)	0.005	4.31 (1.39–13.39)	0.012
Kidney disease	2.60 (1.24–5.42)	0.011	2.59 (1.27–5.27)	0.009
Heart disease	1.67 (1.08–2.57)	0.021	2.00 (1.31–3.04)	0.001
Hypertension	–	NS	–	*
Diabetes	–	NS	–	*
Age > 65 years × hypertension × diabetes	–	*	1.50 (1.02–2.19)	0.039
Model summary				
BIC	1,417.28	–	1,467.84	–
BS	0.191	–	0.201	–
AUC	0.654	–	0.588	–

AUC = area under the curve: measure of discriminatory performance where higher values indicate better performance; BIC = Bayesian information criterion: metric used for the comparison of regression models; BS = Brier score: metric used for overall model performance in which scores range from 0 for a perfect model to 0.25 for a non-informative model; NS = nonsignificant; OR = odds ratio. Data are given as OR and 95% CI.

* Not included in the model.

## DISCUSSION

COVID-19 mortality has been high among critically ill patients and varies widely according to country; population characteristics including biological, environmental, and socioeconomic factors; social isolation policies; and health-system capacity. As other reports, older age and people with kidney and heart diseases were at a higher risk of death.^1,3,4^ In addition, this cohort identified some novel patient-level factors associated with COVID-19 mortality, including infectious diseases coinfection and the interaction between older age and combined hypertension and diabetes.

In Brazil, one in five Brazilian adults has two or more comorbidities^5^ which may increase the susceptibility to COVID-19 mortality. The pivotal link between SARS-CoV-2 and downregulation of angiotensin-converting enzyme-2 in people with multiple comorbidities especially diabetes and hypertension may be a critical role in the overproduction of pro-inflammatory cytokines and COVID-19 severity.^6^ In addition to the relationship between older age and comorbidities, people in poor communities are less likely to have access to health care and social protection, and live in crowded homes with inadequate sanitation and hygiene, which increase the risk for COVID-19 transmission and death.^2^

Although only 1.0% of patients were coinfected with some infectious disease and COVID-19, there was an increased risk of death. Some infectious diseases can cause increased levels of inflammatory cytokines.^7^ The dysregulated immune response in individuals with severe COVID-19 seems to be associated with a cytokine storm. However, the interaction between COVID-19 and other infectious diseases is highly complex, and further epidemiological and immunological studies should be performed.

Although we could not assess effects of other important variables, such as laboratory findings, respiratory support, chronic medications, and acute organ injuries during hospitalization, this large cohort provides data on potential factors associated with COVID-19 mortality from a poor area in Brazil. As risk factors may vary across populational groups, further studies in low- and middle-income cities should be performed to better understand what factors are associated with COVID-19 mortality.
